# Evidence for potential elimination of active *Taenia solium* transmission in Africa?

**DOI:** 10.1056/NEJMc1909955

**Published:** 2020-07-23

**Authors:** S. Gabriël, K.E.M Mwape, P. Dorny

*Taenia solium* taeniosis/cysticercosis is the most important foodborne parasitic zoonosis, affecting over 50 million people and severely impacting public health, social and economic sectors^1^. An integrated human and pig intervention program recently eliminated *T. solium* transmission in Peru^2^. This provided important proof of concept; however, no similar elimination studies have been completed to date in sub-Saharan Africa, where prevalence and infection pressure are higher, and socio-economic contexts are more precarious^3^.

Within ‘CYSTISTOP’ we conducted a two-year integrated human- and pig-based intervention trial in eastern Zambia, to evaluate the feasibility of *T. solium* elimination in a hyper-endemic sub-Saharan African setting. Porcine cysticercosis (primary outcome measure, by carcass dissection) and human taeniosis (secondary outcome measure, by copro-antigen ELISA) prevalence at baseline and post-intervention were assessed in eight intervention villages (1084 people, 184 pigs at baseline), and compared to a negative control group (seven villages, 1329 people, 290 pigs at baseline). Six interventions delivered anthelmintic to humans (praziquantel, 10 mg/kg) and pigs (oxfendazole, 30 mg/kg), pig vaccination (TSOL18 recombinant vaccine, 1 ml) and health education at four-monthly intervals between March 2015 and December 2017, details are available in the protocol including SAP at nejm.org. In the negative control area, only annual health education was implemented. This intervention package was selected as it demonstrated the highest probability of achieving elimination in the ‘cystiSim’ agent-based simulation model for *T. solium*3. Sample size calculations of 34-40 animals per study arm were based on an 80% power to detect an 80% reduction in prevalence, assuming an initial prevalence of 25-30%, using a one-sided likelihood ratio test at the 5% significance level. The effect on prevalence of porcine cysticercosis and taeniosis was estimated using a generalized linear mixed model for binomial data implemented in a Bayesian framework.

Average treatment coverage of eligible human and pig populations was 93.5% and 86.0%, respectively. Average prevalence of viable porcine cysticercosis in the intervention and negative control villages was 32% at baseline, compared to 0% in the intervention villages and 25% in the negative control villages at post-intervention (P<0.001). Taeniosis prevalence in the elimination villages decreased from 16% at baseline to 2% at post-intervention (P<0.001).

The integrated human- and pig-based interventions achieved elimination of viable infection in the pig host and significantly reduced *T. solium* taeniosis prevalence in the study villages. Our findings provide evidence that elimination of *T. solium* transmission may be possible under sub-Saharan African conditions, using the One Health approach.

**Figure:**
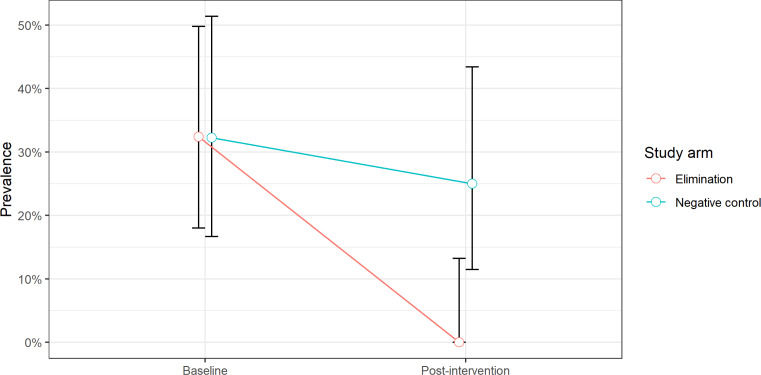


## Supplementary Material

Click here for additional data file.
