# Supplementary material for: Evidence for potential elimination of active Taenia solium transmission in Africa?
Source: N Engl J Med. Author manuscript; Available in PMC 2020 Jul 23. (PMC7289521; doi:10.1056/NEJMc1909955)
Supplement: Supplementary file 1 [file NEJM-2020-1909955-s1.pdf]

Supplementary appendix 1.

This appendix has been provided by the authors to give readers additional information about their work.

Supplement to:

Evidence for potential elimination of active *Taenia solium* transmission in Africa ?

S. Gabriël, K.E.M Mwape*, P. Dorny*

*equal contribution

[1]Ghent University, Ghent, Belgium
[2]University of Zambia, Lusaka, Zambia
[3]Institute of Tropical Medicine, Antwerp, Belgium

And:

Emma C Hobbs Ph.D.[1,3,4], Brecht Devleesschauwer Ph.D.[1, 5], Inge Van Damme Ph.D.[1], Gideon Zulu M.D., M.P.H.[6], Mwelwa Chembensofu B.Sc.[2], Chishimba Mubanga M.Sc.[2], Maxwell Masuku[2], Moses Mambwe B.Sc.[6], Tine De Coster D.V.M.[1], Isaac Khozozo Phiri Ph.D.[2], Dirk Berkvens Ph.D.[3], Angie Colston Ph.D.[7], Emmanuel Bottieau Ph.D.[2], Niko Speybroeck Ph.D.[8], Jennifer Ketzis Ph.D.[4], Arve Lee Willingham Ph.D.[4], Chiara Trevisan Ph.D.[3]

[1] Ghent University, Ghent, Belgium
[2] University of Zambia, Lusaka, Zambia
[3] Institute of Tropical Medicine, Antwerp, Belgium
[4] Ross University School of Veterinary Medicine, Basseterre, St Kitts, West Indies
[5] Sciensano, Brussels, Belgium
[6] Ministry of Health, Lusaka, Zambia
[7] Global Alliance for Livestock Veterinary Medicines (GALVmed), Nairobi, Kenya
[8] Université catholique de Louvain, Institute of Health and Society (IRSS), Brussels, Belgium

* Corresponding author: sarah.gabriel@ugent.be

Table of Contents

1. SUPPLEMENTARY INFORMATION ON THE METHODS INCLUDING THE STATISTICAL ANALYSES: 3
1.1. STUDY OVERVIEW 3
1.2. INTERVENTION DELIVERY 3
1.2.1. HUMAN TREATMENT 3
1.2.2. OXFENDAZOLE TREATMENT AND VACCINATION OF PIGS 4
1.2.3. HUMAN HEALTH EDUCATION 4
1.3. OUTCOME MEASUREMENTS 5
1.3.1. PRIMARY OUTCOME ASSESSMENT BY CARCASS DISSECTION OF PIGS 5
1.3.2. SECONDARY OUTCOME ASSESSMENT BY HUMAN STOOL SAMPLING (ELIMINATION ARM ONLY).. 6
1.4. STATISTICAL ANALYSIS 6
1.5. STUDY OVERSIGHT 7
2. SUPPLEMENTARY INFORMATION ON THE RESULTS 8
2.1. PRIMARY OUTCOME MEASURE – PREVALENCE OF PORCINE CYSTICERCOSIS (BOTH STUDY ARMS).... 8
2.2. SECONDARY OUTCOME MEASURE – TAENIOSIS PREVALENCE (ELIMINATION STUDY ARM ONLY)...... 8
3. ACKNOWLEDGMENTS 8
4. REFERENCES 9

1. Supplementary information on the methods including the statistical analyses:

1.1. Study overview

'CYSTISTOP' is a prospective, large-scale community-based interventional project being conducted in the neighboring Katete and Sinda districts in the Eastern Province of Zambia, where poor sanitation and free-roaming pigs are common. The study areas were selected based on previous epidemiological studies in the region, occurrence of risk factors, accessibility throughout the year and community willingness to participate.

In the 'elimination study arm' (8 villages, 1084 people, 184 pigs at baseline), intensive human- and pig-based interventions including health education were implemented at high frequency, endeavoring to achieve elimination within two years. In the 'negative control study arm' (7 villages, 1329 people, 290 pigs at baseline), only annual health education was conducted.

1.2. Intervention delivery

Briefly, mass anthelmintic administration to eligible humans (praziquantel, 10 mg/kg, or niclosamide 2 g if participant tested seropositive for cysticercosis at baseline) and pigs (oxfendazole, 30 mg/kg), pig vaccination (TSOL-18 vaccine 1 ml) and human health education were conducted in all elimination villages every four months for a total of six iterations. This intervention was selected as it demonstrated the highest probability of achieving elimination in the 'cystiSim' agent-based simulated model for *T. solium* (1). Human health education was delivered in the negative control study arm annually for a total of three iterations.

1.2.1. Human treatment

Mass drug administration was conducted using praziquantel at 10 mg/kg per os, which is the standard treatment for taeniosis in Zambia. For the 86 individuals (7.8% of total population) who tested seropositive for cysticercosis by B158/B60 antigen ELISA at baseline (data not shown), niclosamide was given at standard doses (1 g for children five to six years of age, 2 g for adults and children aged

seven years and above). Niclosamide has a lower efficacy than praziquantel (nearly 80% versus 95-100%) but does not have cysticidal activity and is therefore recommended for treatment of taeniosis in people who also have cysticercosis (2).

Eligibility criteria for anthelmintic treatment were being at least five years of age, not epileptic or seriously ill, living in the study area, and being willing and able to sign the informed consent form and take the treatment.

Anthelmintic therapy was given as direct observed treatments by qualified medical personnel and under the supervision of a medical doctor. Human study participants were monitored by the team's medical doctor for three days after the interventions in case of adverse reactions.

1.2.2. Oxfendazole treatment and vaccination of pigs

All eligible pigs were weighed, and administered an oral drench of oxfendazole at a dose rate of 30 mg/kg (Paranthic™ 10%, MCI Sante Animale, Morocco), as well as 1 mL of TSOL18 vaccine (CYSVAX™, Indian Immunologicals Limited, India) by intramuscular injection into the neck. Oxfendazole is considered to be 100% effective at destroying *T. solium* cysticerci, but degenerating cysts may take between three to six months to completely disappear from tissues (3, 4). TSOL18 vaccine is 99.5-100% effective at preventing porcine cysticercosis, providing pigs have received two doses of vaccine at an interval of no more than five months (5, 6).

Eligible pigs were those aged two months or older, not clinically ill or within four weeks of farrowing, and whose owners had signed the informed consent form.

Treated/vaccinated pigs were ear-tagged and identified with their unique identification code, which included a tally system to record the number of vaccine doses received over the course of the intervention period. All pig handling and treatments were performed by qualified veterinary personnel.

1.2.3. Human health education

Educational activities included village-based sensitization sessions, *T. solium* life cycle posters displayed at the rural health centers, simple informational leaflets distributed to every household during registration, and educational workshops in primary schools using the computer-based cysticercosis advocacy program ‘The Vicious Worm’ (7,8).

1.3. Outcome measurements

Sampling conducted in the study arms at baseline (pigs/humans) and post-intervention (pigs/human) evaluated intervention success. Pigs were used as sentinel animals due to high turnover in study villages (generally slaughtered in their first year of life). Porcine cysticercosis prevalence was the primary outcome measure, determined by the ‘gold standard’ carcass dissection technique performed on slaughter-aged pigs. Humans taeniosis prevalence, determined by coproAg ELISA was the secondary outcome measure.

1.3.1. Primary outcome assessment by carcass dissection of pigs

The sampling strategy for pigs at baseline was one eligible slaughter-aged pig per pig-keeping household. Due to the limited pig numbers at post-intervention, between one and four pigs were selected from pig-keeping households.

Pigs were euthanized by qualified veterinary personnel using a captive bolt gun followed by exsanguination. Carcasses were bisected longitudinally, skin and bones removed, and all organs and muscles removed. Incisions of no more than 3 mm apart were made in all predilection sites (masseters, heart, tongue, psoas, diaphragm, neck), other organs, and remaining musculature from half the carcass for identification of cysticerci, including those of the related tapeworm species *Taenia hydatigena* if present. If no cysticerci were identified in the first half of a carcass, the second half was subsequently dissected.

Numbers, locations and developmental stages of all cysticerci were recorded, and if present a selection of muscular cysticerci, cysticerci located in organs or other unusual locations, and any

suspected cysticerci were excised and collected into individual, labelled tubes for transport to Belgium for analysis. Molecular speciation of cysticerci was conducted by polymerase chain reaction followed by restriction fragment length polymorphism (PCR-RFLP), as described by (9).

1.3.2. Secondary outcome assessment by human stool sampling (elimination arm only)

At baseline, stool samples for taeniosis testing were requested from one adult and one child (if applicable) per household, whereas for the post-intervention sampling, samples were requested from all eligible people. Stool sampling was conducted by distributing labelled sample bottles in small black plastic bags to recruited individuals.

Participants were asked to fill at least half the sample bottle with stool and to return the sample to the study team either the same day or the following morning.

Human stool samples were individually divided into formalin and ethanol aliquots, and stored at room temperature (formalin) or 4°C (ethanol) until transport to Lusaka for analysis. Samples on formalin were subjected to the polyclonal antibody-based copro-AgELISA, for detection of *Taenia* tapeworm antigens in feces (10).

1.4. Statistical analysis

To determine the number of pigs for post mortem sampling it was calculated that 34 to 40 animals would be required per study arm. The objective was to show a reduction in prevalence rather than absence of disease, as the latter required a sample size greater than the actual pig populations in the study areas. The calculations were based on an 80% power to detect an 80% reduction in prevalence, and assuming an initial prevalence of 25-30%, using a one-sided likelihood ratio test at the 5%

significance level. Calculations were performed using SAS 9.3, with the TWOSAMPLEFREQ command in the PROC POWER procedure.

Data were double-entered into EpiData (http://www.epidata.dk/, v3.1) or EpiCollect5 (http://www.epicollect.net/). The effect on the prevalence of porcine cysticercosis was estimated using a generalized linear mixed model for binomial data. Covariates included study arm (negative control versus elimination), study time point (baseline versus post-intervention), and the interaction between both, representing the effect of the elimination program controlled for random fluctuations in prevalence. Study village was included as random effect. Likewise, the effect on the taeniosis prevalence was estimated using a generalized linear mixed model for binomial data with study time point as covariate and study village as random effect.

All models were implemented in a Bayesian framework using Stan (11) via the brms package for R 3.5.1 (12, 13). For each model, four chains of 2000 iterations were run, of which the first 1000 were discarded as warm-up. Convergence was ascertained by inspecting density and trace plots, and by numerical verification that the potential scale reduction factor was below 1.1. The posterior distributions were summarized by their median and a 95% uncertainty interval defined as the distribution's 2.5th and 97.5th percentile. Two-tailed pseudo P-values for coefficients were calculated as $2 * \min[\Pr(\beta < 0), \Pr(\beta > 0)]$.

1.5. Study oversight

The University of Zambia Biomedical Research Ethics Committee (004-09-15, covering both human and animal ethical clearance) and the Ethical Committee of the University of Antwerp, Belgium (B300201628043, EC UZA16/8/73) gave ethical clearance for CYSTISTOP. Approval was also given by local district health authorities, area chiefs and village headmen. The study was introduced and explained at both village and household levels prior to each field visit. Voluntary informed written consent was obtained from all study participants, or written consent from a parent or guardian and

oral assent for children under 18 years of age. Participants were free to withdraw from the study at any time. The funding bodies had no influence over the collection, analysis or interpretation of data, or in writing the manuscript. All authors vouch for the completeness and accuracy of the data and analyses and for the fidelity of the study to the protocol, which is available with the full text of this article at NEJM.org.

Trial registration number: NCT02612896

2. Supplementary information on the RESULTS

2.1. Primary outcome measure – prevalence of porcine cysticercosis (both study arms)

At baseline, viable cysticerci were detected in 32% of dissected carcasses (active porcine cysticercosis) in both study areas. In the elimination arm a prevalence of 32.4% (CI 18.0 – 49.8) was estimated, in the control study arm 32.2% (CI 16.7 – 51.4).

At post-intervention there was a significantly higher reduction in the prevalence of viable cysticerci in the elimination arm compared to the negative control arm (0.0% (CI 0.0 – 13.2) versus 25.0% (CI 11.5 – 43.4), P<0.001).

2.2. Secondary outcome measure – taeniosis prevalence (elimination study arm only)

Taeniosis prevalence in the elimination study arm decreased from 16% (39/251, 95% exact confidence intervals (CI) 11.3 – 20.6%) at baseline to 2.3% (11/480, 95% CI 1.1 – 4.1%) at post-intervention. The change in prevalence was highly significant (P<0.001).

3. Acknowledgments

This study received financial support from the Institute of Tropical Medicine (ITM), Antwerp via the Flemish Ministry of Research (www.ewi-vlaanderen.be/en/science-technology-and-innovation-sti-flanders/funding-rd) and via the Belgian Cooperation in the framework of the collaboration between the ITM and the University of Pretoria, South Africa. Ross University School of Veterinary Medicine provided PhD funding. The praziquantel used in the study was in part provided free of charge by the World Health Organization.

Part of the research was funded by the Bill & Melinda Gates Foundation (https://www.gatesfoundation.org/) and the UK Government through the Global Alliance for Livestock Veterinary Medicines (https://www.galvmed.org/). The findings and conclusions contained within are those of the authors and do not necessarily reflect positions or policies of the Bill & Melinda Gates Foundation nor the UK Government.

Disclosure forms provided by the authors are available with the full text of this article at NEJM.org.

We would like to thank all the field assistants, nurses, students and volunteers who helped with the fieldwork. We are also grateful for the participation of the area chiefs, village headmen and inhabitants of our study villages. Thanks also to Anke Van Hul and Sandra Vangeenberghe for performing the PCR analyses.

4. References

1. Gabriël S, Mwape KE, Phiri IK, Devleesschauwer, Dorny P. *Taenia solium* control in Zambia: The potholed road to success. Parasite Epidemiol Control. 2019;4:e00082.

2. Carabin H, Traoré AA. *Taenia solium* taeniasis and cysticercosis control and elimination through community-based interventions. Curr Top Med Rep. 2014.

3. Gonzalez AE, Gavidia C, Falcon N, Bernal T, Verastegui M, Garcia HH, et al. Protection of pigs with cysticercosis from further infections after treatment with oxfendazole. Am J Trop Med Hyg. 2001;65(1):15-8.
4. Sikasunge CS, Johansen MV, Willingham III AL, Leifsson PS, Phiri IK. *Taenia solium* porcine cysticercosis: Viability of cysticerci and persistency of antibodies and cysticercal antigens after treatment with oxfendazole. Vet Parasitol. 2008;158(2008):57-66.
5. Lightowlers MW, Donadeu M, Elaiyaraja M, Maithal K, Kumar KA, Gauci CG, et al. Anamnestic responses in pigs to the *Taenia solium* TSOL18 vaccine and implications for control strategies. Parasitology. 2016;143(4):416-20.
6. Lightowlers MW. Control of *Taenia solium* taeniasis/cysticercosis: past practices and new possibilities. Parasitology. 2013;140(13):1566-77.
7. Hobbs EC, Mwape KE, Van Damme I, Berkvens D, Zulu G, Mambwe M, et al. Preliminary assessment of the computer-based *Taenia solium* educational program 'The Vicious Worm' on knowledge uptake in primary school students in rural areas in eastern Zambia. Trop Med Int Health. 2018;23(3):306-14.
8. Hobbs EC, Mwape KE, Devleesschauwer B, Van Damme I, Krit M, Berkvens D, et al. Effects of 'The Vicious Worm' educational tool on Taenia solium knowledge retention in Zambian primary school students after one year. PLoS Negl Trop Dis. 2019 May 20;13(5).
9. Dermauw V, Ganaba R, Cissé A, Ouedraogo B, Millogo A, Tarnagda Z, et al. Taenia hydatigena in pigs in Burkina Faso: a cross-sectional abattoir study. Vet Parasitol. 2016;230:9-13.
10. Praet N, Verweij JJ, Mwape KE, Phiri IK, Muma JB, Zulu G, et al. Bayesian modelling to estimate the test characteristics of coprology, coproantigen ELISA and a novel real-time PCR for the diagnosis of taeniasis. Trop Med Int Health. 2013 May;18(5).
11. Carpenter B, Gelman A, Hoffman MD, Lee D, Goodrich B, Betancourt M, et al. Stan: A Probabilistic Programming Language. J Stat Softw. 2017;76(1):32.

12. 25. Buerkner P-C. brms: An R package for Bayesian multilevel models using Stan. J Stat Softw. 2017;80(1):28.
13. 26. R Development Core Team. R: A language and environment for statistical computing Vienna, Austria: The R Foundation for Statistical Computing; 2011 [Available from: http://www.R-project.org/].
